# Incorporating Quaternary Prevention: Understanding the Full Scope of Public Health Practices in Sexual Abuse Prevention

**DOI:** 10.1177/0306624X211049204

**Published:** 2021-10-10

**Authors:** Kieran McCartan, Hazel Kemshall

**Affiliations:** 1 University of the West of England, Bristol, UK; 2 De Montfort University, Leicester, UK

**Keywords:** quaternary prevention, sexual offending, crime, EpiCrim, risk management

## Abstract

This discussion piece argues for a refinement in our understanding of prevention in sexual abuse, suggesting that we include quaternary prevention on the grounds that this concept from medical literature has potential and helpful application to criminal justice and particularly to work with those who cause sexual harm. Located within the paradigm of Epidemiological Criminology (EpiCrim), quaternary prevention extends the prevention spectrum to enable a stronger distinction between tertiary level responses and long-term safe, sustainable reintegration into communities, particularly of those who sexually abuse others. The key principles of quaternary prevention are adapted and refined from current medical literature, and the potential usefulness of quaternary prevention to crime and sex abuse prevention is explored.

## Introduction

Sexual abuse is a complex and multi-faceted issue, as are the reasons why people sexually offend. There has been a growing academic and policy recognition that both understanding and responding to sexual abuse requires a multi-disciplinary approach spanning several different academic disciplines (e.g., psychology, sociology, medicine, criminology). This makes responding to, and preventing, sexual offending challenging, as there is no “one size fits all” approach. Rather what is needed is a dynamic, multi-level response that addresses the individual, inter-relational, community, and society levels (Tabachnick, 2013). Such responses arguably require a pan-disciplinary approach through which differing disciplines can jointly focus on solving a complex problem such as child sexual abuse.

However, current responses to sexual offending across the globe are largely focused on the individual, and the inter-relationship of the individual to families and networks (McCartan, Uzieblo et al., 2021). This has resulted in the person who has committed the offence often being the sole subject of our response, rather than taking a more holistic approach that incorporates society as well as the individual. Sexual offences are collectively as well as individually defined and framed, and therefore, we also need to collectively construct our responses to them including prevention strategies (McCartan et al., 2015). The aim of this discussion article is to open up a discussion about the contribution of Epidemiological Criminology (EpiCrim) to the continued development and relevance of prevention; and to introduce the concept of quaternary prevention from medicine based on the belief that quaternary prevention could have a useful relevance to the criminal justice arena, and particularly to sexual abuse prevention.

## EpiCrim: The Intersection of Public Health and Criminology

**Figure 1.**
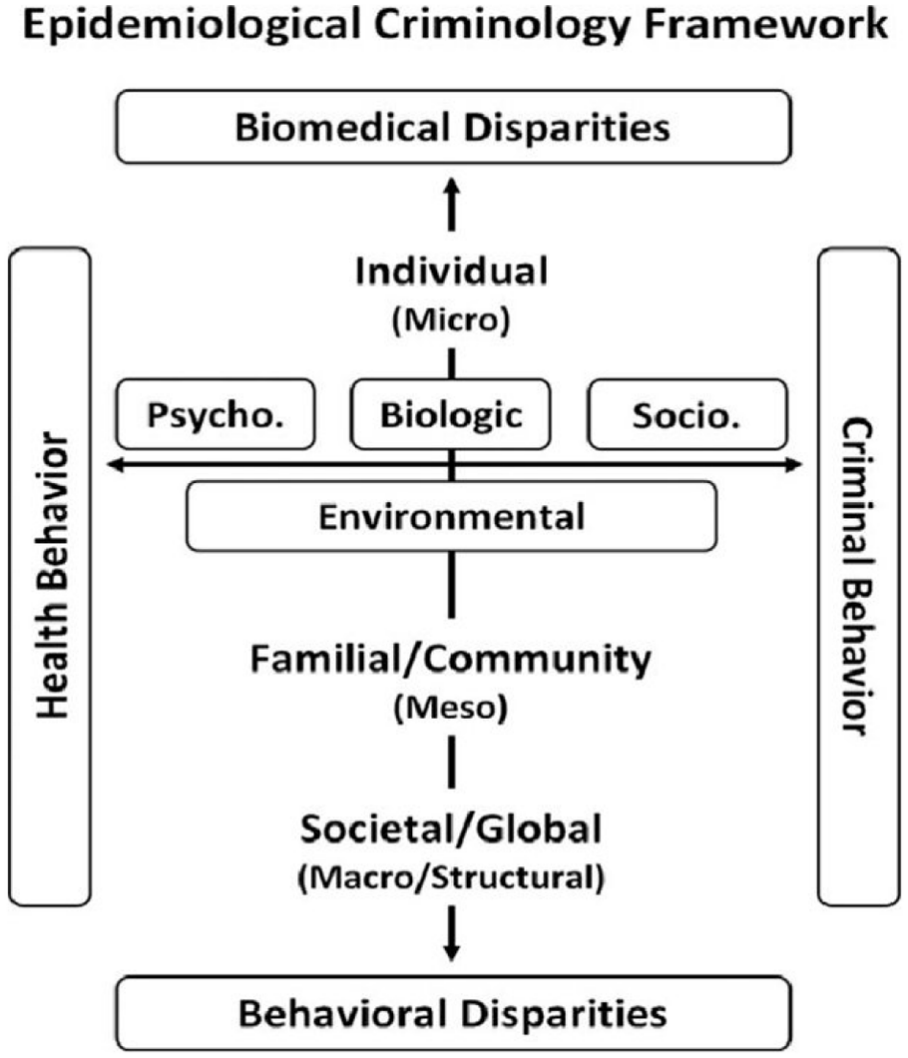
An epidemiological criminology framework (Potter et al., 2012).

Criminology has been described as a “rendezvous subject,” that is a discipline where other social science disciplines interact to focus on complex and multi-faceted issues such as crime (Downes & Rock, 2011). Young (2003) famously described it as being: “*on the busy crossroads of sociology, psychology, law and philosophy*” (p. 97). By the 1980s and 90s the increased “melding” together of different disciplines to understand crime had begun; initially sociology, politics, and psychology, but by the early 2000s health. By the turn of the century public health in particular began to make an important contribution to understanding crime causation (Lainer, 2010). Notable research in this area has included crime causation, life-course analyses of offending behavior, and preventative measures to reduce crime (Akers & Lanier, 2009 ; Lainer, 2010; Lanier & Henry, 2010). This resulted in a new field of research and practice called *Epidemiological Criminology (EpiCrim)* (Lainer, 2010) (see Figure 1). EpiCrim is a recognition that the fields of criminology and health, especially public health, come together in allowing us to understand the causes and consequences of offending behavior better (Lainer, 2010), and this includes sexual offending (McCartan & Prescott, 2020; Waltermaurer & Akers, 2013). In addition, EpiCrim reinforces the life course perspective when analysing and responding to crime. EpiCrim has been defined as:

“*the explicit merging of epidemiological and criminal justice theory, methods and practice. Consequently, it draws from both criminology and public health for its epistemological foundation. As such, EpiCrim involves the study of anything that affects the health of a society, be it: crime, flu epidemics, global warming, human trafficking, substance abuse, terrorism or HIV/AIDS*.” (Lainer, 2010, p. 72).

The international growth in sexual abuse prevention research framed within an EpiCrim perspective testifies to its increasing relevance (McCartan & Prescott, 2020; Skvovtsova, 2013; Waltermaurer & Akers, 2013). Its main contributions to criminal justice, and to sexual abuse prevention, are a focus on the issue at population level; data gathering and analysis at macro as well as micro levels including bringing together behavioral, individual, and societal elements; and working across a range of disciplines to enhance understanding and effective responses. Arguably, EpiCrim enables proactive responses focused on communities, populations at risk as well as individuals, utilizing prevention strategies rooted in health education and knowledge application (Tabachnick et al., 2016).

In recent years there has been a growing recognition that sexual offending, like all forms of crime, is a community, developmental, and life course issue (Brown, 2017; Kemshall, 2017a; McCartan et al., 2015; McCartan, Uzieblo et al., 2021). Examining sexual offending from a life course perspective issue means that this behavior is viewed as a product of someone’s experiences, mental health, well-being, individual differences, and personality (Farrington, 2019; McCartan, 2020). The EpiCrim approach also reinforces current practice developments such as the bio-psycho-social model of rehabilitation, desistance supportive interventions, and trauma informed practice (see: Bradley, 2017; Pringer & Wagner, 2020), and aligns to current research into crime causation and desistance (Farrington, 2019; McCartan, 2020; McCartan & Kemshall, 2020). This is also evidenced by an increased focus on developmental factors and their relationship to crime at both policy and practice levels, for example, Adverse Childhood Experiences (ACEs), trauma, resilience, and well-being and their relationship to later offending behavior (see: McCartan, 2020; Public Health Wales, 2015; Scottish Government, 2018). Therefore, offending behavior, especially sexual offending, is an issue that sits at the intersection of public health and criminal justice (Centers for Disease Control and Prevention [CDC], 2015; Goldsteen et al., 2010; Laws, 2000; Letourneau et al., 2014; Lewis & Dwyer, 2018; McCartan & Prescott, 2020; Public Health England, 2019; Shields & Feder, 2016).

An EpiCrim approach revolves around two key assumptions, which are relevant to Criminology in general, and sexual offending specifically. These are:

1. the application of a Socio-Ecological model to enable the understanding of criminal behavior across the full societal and social spectrum; and
2. the need to examine offending, and related behaviors, from a preventive, public health, and developmental perspective.

We will now examine these two key assumptions and how they potentially offer a new way of thinking about the scope and nature of responding to sexual offending.

### The Socio-Ecological Model and Sexual Offending

The Socio-Ecological Model (SEM) is central to the EpiCrim approach as it frames prevention strategies as an interaction between biology and environment on behavior (Colding & Barthel, 2019). It is an integrated approach to understanding social behavior (including, but not limited to, sexual offending), that incorporates nature (biological and innate) and nurture (societal and learned) explanations for human behavior (CDC, 2004a). The Socio-Ecological Model enables both analysis of and an effective response to, social issues by targeting different population levels. This is epitomized by the seminal work of the CDC (2004a, 2006) on individual, inter-relationship, community, and societal levels (see also Colding & Barthel, 2019).

The Socio-Ecological Model has been applied to crime in general (Fisher-Kowalski, 2015), and sexual offending in particular (Smallbone et al., 2008). The use of SEM in understanding and responding to sexual offending has increased in recent years (Brown, 2017; McCartan et al., 2015), as its “tiered” system allows other disciplines to clearly see how they fit into the prevention and response to sexual abuse. Public health approaches in the area of sexual abuse have drawn heavily on SEM, particularly in terms of research and advocacy for more effective responses (see: Brown, 2017; Kemshall & Moulden, 2017; Letourneau et al., 2014).

### A Preventive, Public Health Approach to Sexual Offending

This article cannot fully explore the remit and scope of public health approaches to sexual offending (see Assini-Meytin et al., 2020; Brown, 2017 for a review). In brief, a public health approach is one that works across different populations in society. A public health approach works to prevent and respond to health-related issues that can have short or long-term consequences. Translating a public health approach into the criminal justice arena means that we would look across different population levels to understand what can be done to reduce criminogenic behavior, anti-social attitudes, and increase an offence free life. It is important to recognize that people convicted of a sexual offence have a lower recidivism rates compared to other individuals with a criminal conviction (Hanson et al., 2014), and therefore when we consider prevention we tend to think about the prevention of first time offending and not the prevention of re-offending (i.e., often framed as risk management); but its important that we consider all forms of prevention equally as they are part of the continuum of the individuals pathway into and out of offending behavior.

Public Health preventative strategies were initially classified as primary, secondary, and tertiary by the CDC (2004a, 2004b):

**Primary Prevention**—intervening before health effects occur, through measures such as vaccinations, altering risky behaviours (poor eating habits, tobacco use), and banning substances known to be associated with a disease or health condition.

**Secondary Prevention**—screening to identify diseases in the earliest stages, before the onset of signs and symptoms, through measures such as mammography and regular blood pressure testing.

**Tertiary Prevention**—managing disease post diagnosis to slow or stop disease progression through measures such as chemotherapy, rehabilitation, and screening for complications.

However, Smallbone et al. (2008) have noted that there are practical issues with implementing this three-tier model, not least the conceptual and definitional problems associated with framing and deploying the tiers (see also: Skvovtsova, 2013). Utilizing an EpiCrim approach, Skvovtsova (2013, pp. 36–50) has refined the CDC preventative tiers into a more tightly defined model with increased policy relevance. It is important to note that the programs and interventions mentioned in Table 1 (see below), as well as Table 2, are based on limited research and small scale, often pilot studies, and therefore offer “promising” results for the positive impact of the prevention of sexual abuse.

**Table 1.** Redefined Levels of Child Sexual Abuse Prevention Adapted from Skvovtsova (2013), With the Addition of Suggested Illustrations and Examples.

Prevention level	Definitions	Suggested examples.
Primary	Interventions to prevent sexual victimization taking place. Prevent crime happening in the first place (Skvovtsova, 2013, p. 39).	- “Upstream” programs focused on elimination of events, conditions, situations, or exposure to risk factors (CDC, 2004a, p. 1; Letourneau et al., 2014; Shields & Feder, 2016). - Focus on intervention prior to sexual abuse or criminality taking place, including the identification of susceptible populations (CDC, 2015). - Includes targeted programs of education, public awareness campaigns, bystander education, professional training (Kemshall & Moulden, 2017).
Secondary	Early detection of sexually abusive behavior, or potential for sexually abusive behavior, and response. Early intervention on trauma on victim support services (Skvovtsova, 2013, pp. 44–46).	- Early intervention victim programs including treatment for trauma (Ogloff et al., 2012). - Interventions and programs targeted at perpetrators/offenders, including the promotion of self-disclosure and help seeking (Dunklefeld, Lucy Faithfull Foundation) (Knack et al., 2019)) - Training of key professionals on recognition and response, and in statutory obligations to report and respond. - Bystander programs for public and communities (Fenton et al., 2016).
Tertiary	Responding to perpetration and victimization. Creating the conditions for offence free lives. Preventing cycle of victimization (Skvovtsova, 2013, pp. 47–49).	- Treatment programs and interventions to tackle sexually abusive behavior and its impact. - Interventions and action to enhance and maintain offence free lifestyles (e.g., desistance focused programs). - Development of social capital and recovery capital to aid reintegration and desistance (McCartan & Kemshall, 2020). - Long-term, non-stigmatizing support of offenders (e.g., CoSA), and effective community re-integration as full citizens (Best, 2019; Best et al., 2018). - Long-term support to victims and survivors, including trauma informed interventions and interventions designed to break the “cycle of victimization” (CDC, 2004a; Fang & Corso, 2007). - Capacity enhancing programs for “at risk” communities (Massachusetts Citizens for Children, 2010). - Strategies and interventions focused on reducing opportunities for re-victimization (e.g., community risk management of offenders; Mulit-Agency Public Protection Arrangements; CoSA). - Promoting self-awareness and self-risk management in “at risk” offender population groups (e.g., CoSA; Dunkelfeld).

**Table 2.** Redefined Tertiary and Quaternary Levels of Sexual Abuse Prevention.

Prevention level	Definitions	Suggested examples
Tertiary	Responding to perpetration and victimization. Creating the conditions for offence free lives. Preventing cycle of victimization (Skvovtsova, 2013, pp. 47–49).	- Treatment programs and interventions to tackle sexually abusive behavior and its impact. - Development of social capital and recovery capital to aid reintegration and desistance (McCartan & Kemshall, 2020). - Capacity enhancing programs for “at risk” communities (Massachusetts Citizens for Children, 2010). - Strategies and interventions focused on reducing opportunities for re-victimization (e.g., community risk management of offenders; MAPPA; CoSA). - Promoting self-awareness and self-risk management in “at risk” offender population groups (e.g., CoSA; Dunkelfeld).
Quaternary	Ongoing, supportive interventions that streamline criminal justice responses to reduce risk of sexual offending. Action taken to identify the risk of over punitiveness on a person convicted of an offence, to protect them from harsher managerial regimes, and suggest interventions which are ethically acceptable.	- Interventions and action to enhance and maintain offence free lifestyles (e.g., desistance focused programs). - Long-term, non-stigmatizing support of offenders (e.g., CoSA), and effective community re-integration as full citizens (Best, 2019; Best et al., 2018). - Long-term support to victims and survivors, including trauma informed interventions and interventions designed to break the “cycle of victimization” (CDC, 2004a; Fang & Corso, 2007).

However, whilst the prevention levels have often been presented as quite distinct (Smallbone et al., 2008), in practice they often overlap, and recent comprehensive programs have sought to work across the levels with some success. The *Enough Abuse* campaign in Massachusetts is perhaps a key example of a comprehensive, multi-layered approach rooted in a public health approach working across the three tiers (see Kemshall & Moulden, 2017 for a description; Schober, Fawcett, & Bernier, 2012; Schober, Fawcett, Thigpen et al., 2012; and Massachusetts Citizens for Children, 2010 for evaluations). In addition, program interventions might overlap or repeat across levels, for example Circles of Support and Accountability (CoSA) supporting both the development of self-risk management at secondary level, and longer-term non-stigmatizing support at tertiary level. Self-risk management strategies may begin at secondary level but be further enhanced and sustained by programs at tertiary level overtime—for example programs that support long-term community reintegration and are desistance supportive (Best, 2019; Best & Colman, 2019; Best & Savic, 2015; Veysey et al., 2013). This aligns to Maruna and Farrall’s (2004) concepts of primary and secondary desistance, with primary referring to initial non-offending, and secondary to the formation of a non-offending identity. McNeill (2016) has added the notion of tertiary desistance, meaning the stage of desistance where the individual is recognized by others as a non-offender and genuine acceptance and belonging occur.

In practice, the prevention levels are often more permeable, and arguably this is reflected in the recent development of more comprehensive approaches to both sexual abuse prevention (CDC, 2015; Letourneau, 2017; Letourneau et al., 2014), with growing relevance to crime reduction more generally particularly in the areas of violence reduction, gun crime, and youth crime. Informed by EpiCrim methodologies and public health approaches a number of responses to violence reduction have been initiated globally. For example, Chicago, Los Angeles, and Philadelphia in the USA have utilized EpiCrim methodologies to identify population level data on crime activities and potential causation in order to develop public health informed strategies for crime reduction (see: Rice et al., 2013). Similar approaches have been pursued in the UK, for example, Merseyside (www.merseysidevrp.com); London (www.london.gov.uk/content/londons-violence-reduction-unit); and the Violence Reduction Programme in Scotland (http://www.svru.co.uk/).

We argue that quaternary prevention is a useful addition to prevention levels in that it focuses on the avoidance of harm, reduces the over-focus on individuals and individual responses, and has the potential to move the policy and practitioner gaze onto long-term reintegration and community reintegration.

**Figure 2.**
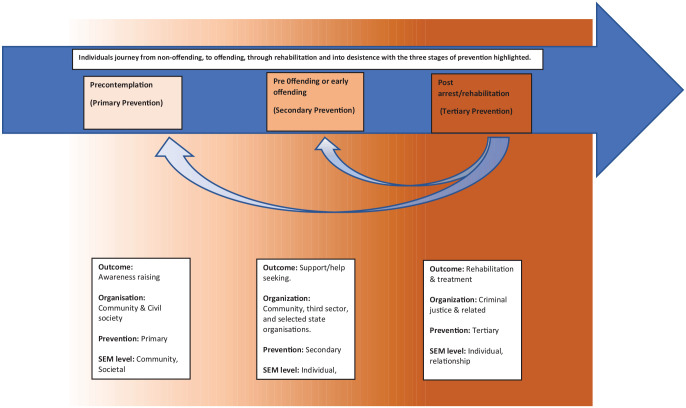
Continuum of sexual offending prevention: the offender’s journey.

Arguably the prevention levels are not actually distinct tiers as suggested in much prevention literature (first coined by Leavell & Clark, 1958; see also: Knack et al., 2019); but rather a continuum with permeable boundaries between the levels, enabling policy, and practice response across different points on the continuum (e.g., Massachusetts Citizens for Children, 2010). The prevention continuum also reflects the idea of the prevention journey for offenders, central to much recent desistance literature (Cid & Martí, 2017; Dufour et al., 2015; Maruna & Mann, 2019); growing life course approaches to public health (see: Public Health England, 2019); and an increased prevention focus on perpetrators of sexual abuse (Assini-Meytin et al., 2020) (see Figure 2).

## The Rise and Relevance of Quaternary Prevention

Quaternary prevention is a relatively new concept derived from the medical field (Tesser, 2017) (although first coined by Jamolle, 1986), defined as:

**“**Action taken to protect individuals (persons/patients) from medical interventions that are likely to cause more harm than good.” (Martins et al., 2018, p. 107)

In brief, it focuses on the prevention of the potential (or actual) over-medicalization and over treatment of patients (Heath, 2013). It is concerned with avoiding over-intervention and focuses on the reduction of harm from actual or potential treatments (Jamoulle, 2012, 2015). It has yet to gain significant impact and use in the sphere of child sexual abuse or crime prevention more generally (see: McCartan, Uzieblo et al., 2021). However, it is arguably a useful concept for both CSA and crime prevention due to its concerns with avoiding harm in the application and use of interventions, and the avoidance of what might be termed “net-widening” (the controversy on the use of statins to prevent high cholesterol is a case in point; Science News, 2017; Tesser, 2017). It is also increasingly concerned with assessing and avoiding future negative events and impacts arising from present actions and interventions (Starfield et al., 2008; Tesser, 2017). Brodersen et al. (2014) focus quaternary prevention on actions taken to protect persons from medical interventions likely to cause more harm than good (cited in Martins et al., 2018).

Whilst the medical quaternary prevention literature considers harm to the patient, rather than the harm posed both to the perpetrator and victim of crime (and sexual crime in particular), quaternary prevention principles and practice are potentially useful to practice with those who offend. Drawing on a range of quaternary prevention literature it is possible to develop a set of key principles currently embedded into medical prevention practice, and adapt them for use in the criminal justice arena. These adapted principles are:

- Do no harm.
- Promotes the importance of harm reduction and the centrality of desistance in reintegration.
- Promotes a bio-psycho-social model to harm reduction.
- Take a service user focused approach that adheres to trauma informed approaches.
- Adopt and critically evaluative stance to all programs, treatments, and interventions.
- Adopt ethical principles for interventions, including robust tests for the limit and extent of interventions, for example, limits and levels of population inclusion in programs and interventions.
- Non-stigmatizing interventions and treatments.
- Avoidance of over-medicalization of issues, problems, communities, and individuals, and in the context of offending avoidance of over-intervention.
- Enables a critical evaluation of and limit to overly precautionary responses (e.g., over-intrusion to prevent all possible risks).
- Evidence-based approach to cost-benefit calculations of prevention.

(Based on: Tesser, 2017; also, Heath, 2013; Jamoulle, 2015; Martins et al., 2018; Sweeney, 2005; Welch et al., 2011).

**Figure 3.**
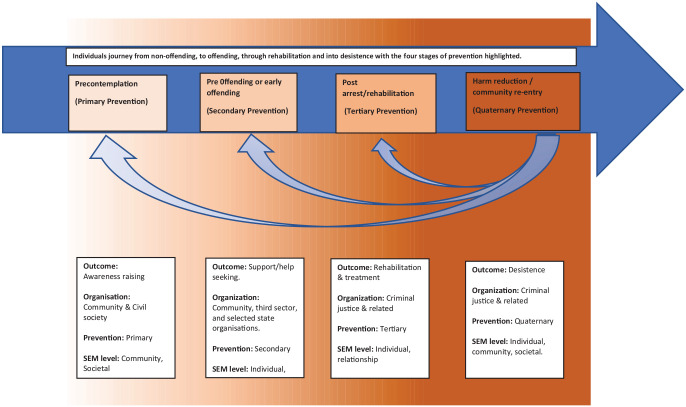
Continuum of sexual offending prevention with the addition of quaternary prevention.

Conceptually, quaternary prevention is best understood not only as an additional prevention level per se, but as a mechanism for quality assuring interventions, treatments, and programs offered at the primary, secondary, and tertiary tiers, particularly against the criteria of limiting harms deriving from interventions themselves (Martins et al., 2018). There is potential for the above principles to be applied to prevention not only in child sexual abuse but also within the crime arena more generally (see Figure 3).

Quaternary prevention has potential relevance to criminal justice, with over-criminalization substituted for over-medicalization. The perils of over-criminalization have been identified in overly punitive responses (Kelly, 2018; The Crime Report, 2020), particularly for persons whose life chances, rehabilitation, and community re-integration are potentially undermined by criminal justice responses (e.g., stigmatization or exclusion). The collateral consequences of penal and criminal justice approaches, particularly to people convicted of a sexual offence, but also to other offenders, are well documented (see McCartan, 2020, and McCartan, Uzieblo et al., 2021). These include responses aimed at either risk reduction or prevention (e.g., registration and disclosure of information relating to people convicted of a sexual offence, Harris, 2017; Kemshall, 2019; McCartan, Harris et al., 2021; Tewksbury & Zgoba, 2010). Internationally we are seeing increasingly punitive criminal justice systems, resulting in over punishment that is potentially problematic (Kelly, 2018). This is relevant to people convicted of a sexual offence because of the nature of their offence, related risk factors, public perception, and current government policies (please see McCartan, 2020 for a broader debate on this issue). Research indicates that these individuals feel more controlled by the criminal justice system, less listened to by professionals, and powerless (McCartan, Harris et al., 2021). Arguably this can undermine the potential for desistance as agency and self-management are seen as critical components of success (Best, 2019; Best & Savic, 2015; Graham & McNeill, 2017; Rocque, 2017; Weaver & Barry, 2014).

Tertiary prevention focuses on immediate responses to sexual abuse including treatment programs and direct interventions (see Knack et al., 2019). The addition of quaternary prevention enables a clear policy and practice focus on longer-term harm reduction strategies and community integration approaches. The distinction between tertiary and quaternary also reflects the actual lived experience of offenders on the prevention journey where the passage from immediate and often intensive interventions to full community integration can be challenging and often unachieved (Best & Savic, 2015). The distinction also enables a greater focus on the differing services, resources, and responses that are required at quaternary level, and could arguably gain increased policy and practice focus for this part of the prevention continuum. Tertiary and quaternary prevention levels are re-defined in Table 2 below:

## Considerations for How Quaternary Prevention Expands Sexual Abuse Relapse Prevention

Genuine and meaningful integration back into communities as full citizens remains problematic for many offenders, particularly for people convicted of a sexual offence (Harris, 2017, 2021) and arguably receives less long-term resource than secondary and tertiary level interventions (McCartan, Merdian et al., 2018). Robust arguments for focusing on pre-perpetration prevention have been made in recent years (e.g., Assini-Meytin et al., 2020); and shifting the focus to “at risk” populations (see e.g., Knack et al., 2019). However, challenges remain at the other end of the spectrum, particularly on interventions and actions to enhance and maintain offence free life-styles, particularly post-sentence. Long-term support to victims and survivors, including trauma informed interventions and interventions designed to break the “cycle of victimization” has also been seen as critical to sexual offence prevention (CDC, 2004a; Fang & Corso, 2007; Saied-Tessier, 2014). Arguably, adding quaternary prevention to the prevention agenda enables a greater focus on safe and constructive long-term re-integration of ex-offenders into the community. It also stretches the policy and practice gaze beyond immediate re-entry concerns toward long-term sustainability of offence-free lives, and to actions which promote full participation into communities rather than merely being placed within them (Best, 2019; Best & Savic, 2015). A focus on long-term re-integration would require consideration of the “recovery capital” necessary to achieve sustained re-integration (McCartan & Kemshall, 2020), and steps to mitigate the stigma and rejection experienced, particularly by those who have offended against children (Harris, 2021; McCartan & Gotch, 2020). This has been labeled as “negative social capital,” contributing strongly to the outsider status of many ex-offenders (Best & Savic, 2015). In this sense, quaternary prevention can be understood as the maintenance and reinforcement period aimed at consolidating tertiary level interventions.

The underlying principle of quaternary prevention is “do no harm” and in this context, avoid over-punitive responses. This is particularly challenging for responses to sexual offenders given public concern, prevailing precautionary principles, and media coverage which can result in long-term management strategies which are over-intrusive and constraining (Kemshall, 2017a, 2019). Practice and policy strategies which incorporate a demonstrable sense of proportion have been critical to managing such challenges (Kemshall, 2017a, 2017b), coupled with approaches that explicitly seek to balance risks, rights, and reintegration with a clear and evidenced rationale for decisions subsequently made (Kemshall et al., 2013). This response should be the least harm to the individual offender that is possible commensurate with safely managing risk; focusing on safe and supportive reintegrative strategies (Kemshall, 2008). Importantly such an approach would encourage practitioners to explicitly weigh up the extent to which preventive measures were anxiety driven or risk driven (Fenton, 2013), thus avoiding the possibility of over-reaction and promoting defensible decisions not defensive ones (Kemshall, 2009).

Internationally we are starting to see the development of emerging good practice in this area. For example, New Zealand has reframed their register of people convicted of a sexual offence into a pro-active, supportive, strengths-based management tool for sexual offenders, and is an example of quaternary prevention in action (McCartan & Laws, 2018). The New Zealand (NZ) model is located within a treatment and rehabilitation framework, with the aim of the register being to help the community management of people convicted of a sexual offence integrate back into the community. It is constructed as a pro-social welfare and safeguarding tool and not as a punitive, controlling tool. The role of the register in New Zealand is to keep at risk offenders under scrutiny but the different mode and character of delivery ensures that it is constructed in a supportive way that reinforces treatment, desistance, and harm reduction. The principles of the “Good Lives Model” (Purvis et al., 2013), have been incorporated into the NZ register, and embedded into staff training and operational policy (Barnes & Law, 2019). This has resulted in: “strengths-based case management practice which balances the need to manage risk, with the needs of the person on the register” (Barnes & Law, 2019: 27), supported by a commitment that the register should effectively support case management (Masters & Kebbell, 2019; McCartan, Hoggett et al., 2018). This helpfully reframes lifetime supervision as proactive and supportive risk management and not, as it is in other countries, as an exclusionary labeling system that allocates persons to a lifetime of risk stigmatization (for UK possibilities for change see: O’Sullivan et al., 2016). Evaluation of the NZ registry is ongoing.

In addition, quaternary prevention also explicitly seeks to foreground the evidenced consideration of proportionality as well as ethical guidelines for the choice in the implementation of interventions (Brodersen et al., 2014; Martins et al., 2018), both of which are essential in effective risk management. In recent years, there have been considerable strides taken to promote increased ethical practice with “high-risk” and “very high-risk” individuals (Mann et al., 2018; Maruna & Mann, 2019), and notably by Ward et al. (2009), who apply a human rights framework to those sexual offenders receiving compulsory treatment within the criminal justice system (2009; see also O’Loughlin, 2016). Which reinforces the importance of a service user lead, trauma informed approach.

Making, acquiring, and sustaining a new, offence-free identity has also been seen as intrinsic to desistance, including from sexual offending (Harris, 2017, 2021). Avoiding interventions that actively undermine this process can also be understood as “do no harm”; but also requires an evidenced and critical evaluation of precautionary responses aimed at preventing all possible risks (Hebenton & Seddon, 2009; Kemshall, 2017b; McAlinden, 2007, 2010). In particular, sentencing utilizing a “precautionary principle” is seen as contrary to prevailing concerns with individual rights and rule of law (Lippke, 2008). Such sentencing is usually reserved for particular types of offences or offenders (e.g., sexual offenders, terrorists) but has raised more general concerns and spawned an emerging literature on the jurisprudence of the “preventive state” (see Janus, 2006; Krasmann, 2007; Slobogin, 2003, 2011). This has extended into concerns about community based preventative measures particularly for people convicted of a sexual offence, such as registration, and compulsory supervision (McCartan et al., 2017); and is evidenced by the recent terrorism review into sentencing and community surveillance (Hall, 2020). For some commentators, the growth in prevention measures driven by an unrestrained precautionary logic has been described as a “pre-crime society,” “in which the possibility of forestalling risks competes with, and even takes precedence over, responding to wrongs done” (Zedner, 2007, p. 261, see also; Zedner, 2005, 2009). Quaternary prevention offers the possibility of focusing more strongly on responding to (and healing) the harms done—for victims, perpetrators, and communities. Quaternary prevention is an inclusive, restorative, and service user led approach with safe, sustained reintegration as its core aim. It also focuses attention at policy and practitioner levels toward structural and community-based initiatives and reduces the over-focus on an individual response as the sole answer. It also recognizes that total reliance on the criminal justice system to reduce or prevent sexual abuse is not helpful, and that other processes via quaternary prevention have much to offer. However, to implement it we need to change our narrative on responding to sexual abuse. We need to think more about the service user and there journey as apposed targeting our interventions as a response to an incident, or incidences, of sexual abuse.

Maruna (2001) asked us to rethink punishment and rehabilitation and to consider desistance not control, working within a person-centered approach focused on desistance supportive work. Desistance recognizes that managing your own risk of offending is difficult, often referred to as the “pains of desistance” (Nugent & Schinkel, 2016); and that it can take different time periods for each person based on their lived context (Harris, 2017, 2021). Hanson et al. (2014) have argued that achieving desistance in people convicted of a sexual offence is the outcome of positive, pro-social, supportive integration (Harris, 2017, 2021), and arguably a greater focus on quaternary prevention would enable both a policy and practice shift to these activities. The challenge that we face in promoting quaternary prevention in the criminal justice system is the potential for public misperception and political rejection, particularly perceptions about “being soft on sex offenders,” and de-prioritising victims (Harper & Hogue, 2015; Stafford & Vandiver, 2017). Public understandings of sexual abuse tend to be characterized by fearfulness and moral repugnance (Harper et al., 2017; Jahnke, 2018), with the potential benefits of prevention misunderstood or rejected (McCartan, Uzieblo et al., 2021). However, in an era of growing resource constraint post the COVID-19 pandemic harsh cost-benefit decisions about the community management of sexual offenders will have to be made. Cost and effectiveness may therefore play an increasing role in determining prevention responses rather than punishment or public censure (Frost, 2010), and redeemability may become an important public message (Maruna & King, 2009). Punishment, especially custody, and re-integration failures are costly, both economically and socially. For example, the annual cost of providing a prison place in England and Wales is £43,213 (Ministry of Justice, 2019), on probation is £2,380 (Ministry of Justice, 2012), and a prison-based Sex Offender Treatment Program in England and Wales is £8,476 (Brookes et al., 2013). Quaternary prevention focused on successful long-term reintegration and harm reduction could potentially reduce such costs and increase community safety (Saied-Tessier, 2014), mirroring similar trends within the arena of drug use (Stevens, 2011). The route to an offence free life is via harm reduction, re-integration back into the community, and sustained behavior change.

## Conclusion

We have argued in this discussion piece for a refinement in the prevention levels to include quaternary prevention. The rationale for this is to enable a stronger distinction between tertiary level responses and long-term safe, sustainable reintegration into communities, particularly of those who sexually abuse others. The focus on quaternary prevention has the potential to extend the policy and practice gaze to long-term meaningful reintegration of those often the most feared and stigmatized, and focus practice beyond the immediate challenges of re-entry. Quaternary prevention also importantly prioritizes ethical practices, proportionate responses, and evidenced approaches to interventions and management over the long-term. Within the growing context of resource constraint quaternary prevention might just be an idea whose time has come.
